# Volatile Compounds of Young Wines from Cabernet Sauvignon, Cabernet Gernischet and Chardonnay Varieties Grown in the Loess Plateau Region of China

**DOI:** 10.3390/molecules15129184

**Published:** 2010-12-10

**Authors:** Bao Jiang, Zhenwen Zhang

**Affiliations:** 1 College of Enology, Northwest A&F University, Yangling 712100, China; E-Mail: treebaojiang@163.com (B.J.); 2 Shaanxi Engineering Research Center for Viti-Viniculture, Northwest A&F University, Yangling 712100, China

**Keywords:** volatile compounds, young wines, Loess Plateau region, GC–MS

## Abstract

In order to elucidate the aroma components of wine produced in the Loess Plateau region of China, volatile compounds of young wines from Cabernet Sauvignon, Cabernet Gernischet and Chardonnay varieties grown in the new ecological region were investigated for the first time in this research. Among the volatile compounds analyzed by HS-SPME with GC-MS, a total of 45, 44 and 42 volatile compounds were identified and quantified in Cabernet Sauvignon, Cabernet Gernischet and Chardonnay wines, respectively. In the volatiles detected, alcohols formed the most abundant group in the aroma compounds of the three wines, followed by esters and fatty acids. According to their odor active values (OAVs), 18 volatile compounds were always present in the three wines at concentrations higher than their threshold values, but ethyl octanoate, ethyl hexanoate, and isoamyl acetate were found to jointly contribute to 92.9%, 93.3%, and 98.7%, of the global aroma of Cabernet Sauvignon, Cabernet Gernischet and Chardonnay wines, respectively. These odorants are associated with “fruity’’ and ‘‘ripe fruit’’ odor descriptors.

## 1. Introduction

Wine, which is produced by fermentation of fresh grapes or must, is one of the most complex alcoholic beverages, and its aroma substances are responsible for much of this complexity [1]. Wine flavor can be classified into three groups: varietal, fermentative and wine ageing aroma. Describing the aroma of wines is not a simple task for researchers, because more than 800 aroma compounds such as alcohols, esters organic acids, aldehydes, ethers, ketones and terpenes, *etc*., have been identified in them [2], with a wide concentration range varying between hundreds of mg/L to the μg/L or ng/L levels [3], and their combinations form the character of wine and differentiates one wine from another.

Aroma of a wine is one of the major factors that determine its nature and quality [4,5] and it can be influenced by several factors: environment (soil and climate), grape variety, ripeness, fermentation conditions, biological factors (yeast and other components of the oenological microflora), the wine production process and aging [6]. Cabernet Sauvignon and Chardonnay are popular varieties all over the world, and much research had been carried out to study the aroma from the two monovarietal wines during the last few decades [7,8,9], whereas Cabernet Gernischet is a special wine-grape variety only cultivated in Jiaodong Peninsula and Changli County of China, and is used by Chinese wineries to prepare premium quality wines.

Several analytical methods have been used for the extraction and determination of volatile compounds. Previously, volatile aroma analysis relied on distillation, solvent extraction, or concentration on solid-phase supports to isolate and concentrate aroma compounds [10,11,12]. These methods are time consuming, result in extensive solvent use, waste and solvent costs, and can result in losses of some important volatiles depending on solvent selectivity and volatility. In addition, liquid–liquid extractions frequently require heating the sample, which can result in component degradation and artifact formation. Headspace analysis (both static and dynamic) has been widely used for the analysis of grape and wine volatiles [13,14]. However, static headspace analysis often suffers from poor sensitivity for trace volatiles and dynamic headspace analysis suffers from interferences from water and ethanol [15,16]. 

Solid-phase microextraction (SPME) has now widely been used for the analysis of aroma volatiles in many food and beverage matrices in recent years [17]. SPME integrates the extraction, concentration and introduction in one step and the use of SPME results in reducing preparation time and simultaneously increasing sensitivity over other extractions. Therefore, it could be considered as a simple, efficient and environment-friendly sample preparation method, numerous SPME applications for volatiles in wines have been reported [18,19,20].

With the development of the Chinese wine industry, grape-growing areas have been increasing, and new wine-growing regions are being constantly discovered, including the Loess Plateau region of China which occupies about 600 thousand square kilometers. Rongzi Chateau of Xiangning County is located in Loess Plateau region, where the different characteristics of the landform such as crisscross gulleys, different slopes, slope direction and altitude contribute together to form the local mountainous microclimate. The mean annual temperature and that of the coldest month (January) are 9.9 °C and −6 °C, respectively. Active accumulated temperature (≥10 °C) is more than 2,998 °C with proper precipitation (annual rainfall around 50 cm). The climatic characteristics of dryer air, stronger sunshine, and a wide swing in diurnal temperature differences distinguished by lower night-time temperatures create an especially healthy environment for vines in the Loess Plateau region, but to date, there has been no published research on the volatile characteristics of wines produced in the Loess Plateau region of China, and their study could help winemakers optimize operational conditions (harvest parameters, juice preparation, fermentation techniques, use of yeasts, bacteria and enzymes, *etc*.) in order to emphasize one or more aromas in the final wines. In this study, we selected three representative varieties, Cabernet Sauvignon, Cabernet Gernischet and Chardonnay, which are separately cultivated at Rongzi Chateau in Xiangning County, and investigated the volatile compounds of three monovarietial wines, with volatiles being extracted by solid-phase microextraction (SPME) and detected by GC–MS.

## 2. Results and Discussion

The experimental results are given in Table 1. In all 54 volatile compounds were identified in the three wines studied, including 23 alcohols, 13 esters, eight acids, three terpenes and seven aldehyde and ketone compounds. Many of these volatile compounds are commonly found in wines and are derived from grapes and yeast strain fermentation and the vinification process [21]. 

**Table 1:** The threshold values and concentration of volatile components found in the Cabernet Sauvignon, Cabernet Gernischet and Chardonnay young wines.

Compounds	Threshold(μg/L)	Odor descriptor	Concentration (μg/L)		
			Cabernet Sauvignon	Cabernet Gernischet	Chardonnay
**Alcohols **					
1-Propanol	306,000^d^	Fresh, alcohol	ND	2715.9	6491.9
Isobutyl alcohol	40,000^a^	Fusel, alcohol	22004.1	19778.1	9379.0
1-Butanol	150,000^c^	Medicinal, alcohol	1587.6	1083.4	945.2
Isoamyl alcohol	30,000^a^	Cheese	185587.5	144784.9	103021.5
1-Pentanol	80,000^j^	Fruity, balsamic	176.1	146.8	ND
4-Methyl-1-pentanol	50,000^h^	—	106.8	81.0	28.5
2-Heptanol	70^e^	Fruity, mouldy, musty	14.7	8.1	ND
1-Hexanol	8000^a^	Green, grass	4017.7	2542.8	1163.7
3-Hexen-1-ol,(E)-	400^a^	Green, floral	133.8	92.6	55.0
3-Hexen-1-ol,(Z)-	400^a^	Green	135.8	129.2	54.4
2-Hexen-1-ol,(E)-	400^j^	Green grass, herb	47.3	ND	ND
2-Hexen-1-ol ,(Z)-	400^j^	Green grass, herb	ND	ND	16.6
2-Octanol	120^a^	—	24276.6	ND	ND
1-Heptanol	1000^j^	Grape, sweet	212.2	129.0	22.3
2-Ethyl-1-hexanol	8000^j^	Mushroom, sweet fruity	trace	ND	ND
2-Nonanol	58^e^	—	6.3	ND	3.7
2,3-Butanediol,[R-(R*,R*)]-	120,000^i^	Butter, creamy	405.4	475.1	527.9
1-Octanol	120^a^	Intense citrus , roses	44.2	25.2	17.0
3-(Methylthio)-1-propanol	500^a^	Boiled potato, rubber	1990.2	2006.8	ND
1-Decanol	400^a^	Orange flowery, special fatty	10.7	9.4	7.7
Benzyl alcohol	200,000^f^	Citrusy, sweet	150.4	137.8	ND
2-Phenylethanol	14,000^a^	Flowery, pollen, perfumed	14504.8	18164.6	9165.8
1-Dodecanol	1000^i^	Unpleasant at high concentration, flowery at low concentration	897.0	1770.7	ND
Subtotal (μg/L)			256309.2	194081.4	130900.2
Subtotal (%)			64.7	59.2	46.6
**Esters**					
Ethyl acetate	7500^a^	Fruity, sweet	52205.7	69820.2	32920.7
Isobutyl acetate	1600^a^	Solvent	ND	ND	339.4
Isoamyl acetate	30^a^	Banana	14733.8	13999.9	24849.9
Ethyl hexanoate	5^a^	Fruity, anise	4107.2	2985.1	5535.6
Hexyl acetate	670^d^	Pleasant fruity, pear	142.4	144.9	562.6
Ethyl lactate	154,636^c^	Lactic, raspberry	11724.2	8119.4	2223.6
Heptyl acetate	1400^j^	Almond, pear	14143.9	ND	5733.0
Methyl octanoate	200^k^	Intense citrus	8.5	8.5	5.4
Ethyl octanoate	2^a^	Pineapple, pear, floral	5107.9	4284.0	13767.8
Ethyl decanoate	200^a^	Fruity, fatty, pleasant	1451.2	1522.5	7962.0
Diethyl succinate	500,000^c^	Light fruity	182.9	266.8	46.3
Phenethyl acetate	250^a^	Pleasant, floral	ND	37.2	310.4
Ethyl dodecanoate	1500^j^	Flowery, fruity	222.7	264.5	2028.6
Subtotal (μg/L)			104030.4	101453.0	96285.3
Subtotal (%)			26.3	31.0	34.2
**Acids**					
Acetic acid	200,000^a^	Acid, fatty	18901.2	11538.3	23016.1
Propanoic acid	8100^f^	Vinegarish	213.3	907.2	ND
Isobutyric acid	200,000^a^	Fatty	2916.4	2335.3	1798.4
Isovaleric acid	33.4^b^	Fatty, rancid	ND	1961.2	898.0
Hexanoic acid	3000^a^	Cheese, rancid, fatty	1709.8	1155.1	1714.3
Heptanoic acid	3000^g^	Fatty, dry	trace	ND	ND
Octanoic acid	500^a^	Rancid, harsh, cheese, atty acid	5205.9	3848.5	11208.7
Decanoic acid	15,000^a^	Fatty, unpleasant	2877.7	2477.4	12089.7
Subtotal (μg/L)			31824.3	24223.0	50725.2
Subtotal (%)			8.0	7.4	18.0
**Aldehydes and ketones**					
Nonanal	1^l^	Green, slightly pungent	49.0	69.9	18.5
Benzaldehyde	2000^d^	Almond	348.6	95.2	349.9
Geranylactone	60^j^	Floral	29.8	26.4	26.4
β-Ionone	0.09^b^	Violet	ND	5.8	ND
Decanal	1000^h^	Grassy, orange skin-like	ND	2175.0	ND
Furfural	14,100^a^	Pungent	125.9	ND	182.6
Acetoin	150,000^a^	Flowery, wet	3378.5	5595.8	2645.7
Subtotal (μg/L)			3931.8	7968.1	3232.2
Subtotal (%)			1.0	2.4	1.2
**Terpenes**					
Citronellol	100^a^	Green lemon	14.4	14.5	9.4
Linalool	25.2^b^	Fruity, citric	ND	ND	trace
Limonene	200^e^	Flowery, green, citrus	ND	124.3	36.1
Subtotal (μg/L)			14.4	138.8	45.5
Subtotal (%)			<0.1	<0.1	<0.1
**Total**			396110.1	327864.3	281188.4

The data were mean values of triplicate samples (maximum SD: ±10%); ND, Not determined; ^a^ [30]; ^b^ [7]; ^c^ [36]; ^d ^[37]; ^e ^[38]; ^f ^[39]; ^g ^[40]; ^h ^[41]; ^I ^[9], ^j ^[42], ^k^[43], ^l^ [44].

### 2.1. Alcohols

Alcohols represented the largest group in terms of the number and concentration of aroma compounds identified in all three wines, followed by esters and fatty acids. The subtotal concentrations of alcohols in the three wines were in the 130,900.2–256,309.2 μg/L range, being 46.6–64.7% of the total volatile compounds detected. 

Alcohols are formed from the degradation of amino acids, carbohydrates, and lipids [22]. The composition of alcohols differed both qualitatively and quantitatively among the three wines. This volatile fraction was mainly composed of isoamyl alcohol, 2-octanol, isobutyl alcohol and 2-phenylethanol; these four alcohols had concentrations >9,000 μg/L, (and existed in at least one of the wines studied). Isoamyl alcohol was the most abundant alcohol, accounting for *>*72% of the total alcohols in all three wines studied. Cabernet Sauvignon and Cabernet Gernischet wines contained 20 and 18 types of alcohols, respectively, and the alcohol profiles of the two wines were more diverse than that of Chardonnay wine which only contained 15 types of alcohols. (*E*)-2-Hexen-1-ol, 2-octanol and 2-ethyl-1-hexanol were absent in the wine made from Cabernet Gernischet and Chardonnay. Other missing alcohols in Chardonnay wine were 1-pentanol, 2-heptanol, 3-(methylthio)-1-propanol, benzyl alcohol and 1-dodecanol, and in Cabernet Gernischet wine they were (*Z*)-2-hexen-1-ol and 2-nonanol. In Cabernet Sauvignon wine, 1-propanol and (*Z*)-2-hexen-1-ol were absent, and 2-ethyl-1-hexanol was only present in trace amounts. 

### 2.2. Esters

There were also significant differences in the type and amount of esters present in the three wines. In general, the number and proportion of esters in Chardonnay wine (34.2%) were higher than those of Cabernet Gernischet (31.0%) and Cabernet Sauvignon (26.3%), but the total ester contents in the three wines exhibited the opposite order. Although their amounts differed in the three wines, ethyl acetate, isoamyl acetate, ethyl lactate and ethyl octanoate were the major esters found in the aroma compounds. Phenethyl acetate and heptyl acetate were absent in Cabernet Sauvignon and Cabernet Gernischet wines, respectively, while isobutyl acetate was unique to Chardonnay wine. Acetate esters are the result of the reaction of acetyl-CoA with higher alcohols formed from degradation of amino acids or carbohydrates [23]. On the other hand, fatty acid ethyl esters are produced enzymatically during yeast fermentation and from ethanolysis of acyl-CoA formed during fatty acid synthesis or degradation. Their concentration depends on several factors: yeast strain, fermentation temperature, aeration degree and sugar content [23]. Ethyl lactate, a product of malolactic fermentation during wine vinification [24], was lower in Chardonnay wine than in Cabernet Sauvignon and Cabernet Gernischet wines.

### 2.3. Fatty acids

The production of fatty acids has been reported to be dependent on the composition of the must and fermentation conditions [25]. Acetic acid was the major fatty acid found, constituting 45.4% to 59.4% of the total fatty acid content of the wines. Acetic acid is produced during alcoholic and malolactic fermentation. At low levels this compound lifts wine flavors; however, at high levels, it is detrimental to the taste of wine by leaving the wine tasting sour and thin [26]. Heptanoic acid could only be identified as a trace component in Cabernet Sauvignon wine. Isovaleric acid and propanoic acid were absent in Cabernet Sauvignon and Chardonnay wines, respectively. Isobutyric acid, hexanoic acid, octanoic acid, and decanoic acid were found in all three wines. These C_6_ to C_10_ fatty acids at concentrations of 4 to 10 mg/L impart mild and pleasant aroma to wine; however, at levels beyond 20 mg/L, their impact on wine becomes negative [27]. The C_6_ to C_10 _fatty acids might have a positive impact on the aroma of the three wines examined in the current study since their levels were all far below 20 mg/L.

### 2.4. Terpenes

Numerous studies have reported that the terpenoid compounds could be used analytically for varietal characterization. It is known that terpene compounds are secondary plant constituents, whose biosynthesis begins with acetyl- CoA [28]. The formation of terpenes by *Saccharomyces cerevisiae* has not previously been observed and terpenes are not changed by the yeast metabolism during fermentation [29]. In the present study, three terpenes were detected in the wines. They were citronellol, linalool and limonene, and their concentrations were very low. They made up of less than 0.1% of the total volatile compounds in all three wines. Linalool was found solely in Chardonnay wine, the limonene could only be detected in Cabernet Gernischet and Chardonnay wines. Hence, these terpenyl compounds could serve as potential indicators to distinguish wine derived from Chardonnay from those from Cabernet Sauvignon and Cabernet Gernischet [8].

### 2.5. Aldehydes and ketones

The composition of aldehydes and ketones varied greatly between the wines. Nonanal, benzaldehyde, geranylactone and acetoin were found in all three, whereas β-ionone and decanal were unique in the aroma compounds of Cabernet Gernischet wine, and furfural existed only in Cabernet Sauvignon and Chardonnay wines.

### 2.6. Odor activity values (OAVs)

Though dozens of volatiles were detected in each wine sample, not all of the components have the same impact on the overall aroma character of a wine. Of all the compounds analyzed, only those displaying OAVs greater than 1 were deemed to contribute to wine aroma [30]. The contribution of each volatile compound with OAVs above 1 to the aroma of each wine can be evaluated qualitatively by means of its associate descriptor, and quantitatively by means of its OAVs. 

**Table 2:** Odor activity values ^a ^(OAVs) and relative odor contribution ^b^ (ROC) for the aroma compounds in Cabernet Sauvignon, Cabernet Gernischet, and Chardonnay young wines.

Compounds	Odor descriptor	Cabernet Sauvignon	Cabernet Gernischet	Chardonnay
Ethyl octanoate	Pineapple, pear, floral	2554.0 (61.4%)	2142.0 (62.7%)	6883.9 (77.0%)
Ethyl hexanoate	Fruity, anise	821.4 (19.7%)	597.0 (17.5%)	1107.1 (12.4%)
Isoamyl acetate	Banana	491.1 (11.8%)	446.7 (13.1%)	828.3 (9.3%)
2-Octanol	—	202.3 (4.9%)	—	—
Nonanal	Green, slightly	49.0 (1.2%)	69.9 (2.0%)	18.5 (0.2%)
β-Ionone	Violet	—	64.4 (1.9%)	—
Isovaleric acid	Fatty, rancid	—	58.7 (1.7%)	26.9 (0.3%)
Ethyl decanoate	Fruity, fatty, pleasant	7.3 (0.2%)	7.6 (0.2%)	39.8 (0.4%)
Octanoic acid	Rancid, harsh, cheese	10.4 (0.2%)	7.7 (0.2%)	22.4 (0.3%)
Heptyl acetate	Almond, pear	10.1 (0.2%)	—	4.1 (<0.1%)
Ethyl acetate	Fruity, sweet	7.0 (0.2%)	9.3 (0.3%)	4.4 (<0.1%)
Isoamyl alcohol	Cheese	6.2 (0.1%)	4.8 (0.1%)	3.4 (<0.1%)
3-(Methylthio)-1-propanol	Boiled potato, rubber	4.0 (0.1%)	4.0 (0.1%)	-
Decanal	—	—	2.2 (<0.1%)	—
1-Dodecanol	Orange flowery, special fatty	0.9 (<0.1%)	1.8 (<0.1%)	—
Ethyl dodecanoate	Flowery, fruity	0.2 (<0.1%)	0.2 (<0.1%)	1.4 (<0.1%)
2-Phenylethanol	Flowery, pollen, perfume	1.0 (<0.1%)	1.3 (<0.1%)	0.7 (<0.1%)
Phenethyl acetate	Pleasant, floral	—	0.1 (<0.1%)	1.2 (<0.1%)

^a^ OAVs were expressed as the mean concentration of an aroma compound divided by its odor threshold value; ^b ^ROC of each aroma compound shown in parentheses was calculated as the ratio of the OAV of the individual compound to the total OAV of each wine.

Table 2 listed the OAVs for the 18 odor-active compounds (OAV > 1 in at least one of the wines studied), Chardonnay and Cabernet Sauvignon wines had the same odorants with OAVs above 1, and in contrast, Cabernet Gernischet wine contained the most odor-active compounds, suggesting that the Cabernet Gernischet wine presented more complex flavor than the others. In addition, the OAVs for ethyl octanoate, ethyl hexanoate and isoamyl acetate in Chardonnay wine were close to two times higher than those of Cabernet Sauvignon and Cabernet Gernishcet wines.

The computation of relative odor contribution (ROC) proposed by Ohloff [31] is a useful index for determining the important aroma components in a complex system. Based on the ROC values of single compound, we found that the global aroma of all three wines was dominated by fermentative aromas, namely, the ethyl esters of fatty acids that conferred fruity notes to all the wines. Specifically, ethyl octanoate, ethyl hexanoate, and isoamyl acetate jointly accounted for 92.9%, 93.3%, and 98.7%, of the global aroma of Cabernet Sauvignon, Cabernet Gernischet and Chardonnay wines, respectively (Table 2). The results in this study were partially consistent with the results in a previous study [8] showing that the three aroma compounds mentioned above jointly accounted for 97% and 99%, respectively, of the global aroma of Cabernet Sauvignon and Cabernet Gernischet wines from Huailai County of China. In our study, alcohols were the predominant groups which constituted the aroma compounds rather than acids as in the previous study [8]. Thirteen aroma compounds whose concentrations are higher than threshold values from the Chardonnay wine produced in Changli County of China were identified [9]. In contrast, 12 aroma compounds whose concentrations are higher than threshold values were idenified in present study, among which seven aroma compounds, namely ethyl octanoate, ethyl hexanoate, isoamyl acetate, ethyl decanoate, octanoic acid, isoamyl alcohol, and phenethyl acetate overlapped with ones in the study above. The characteristics of local climate and soil could lead to the discrepancy between our results and others. Based on the OAVs and the ROC values of aroma compounds, ethyl octanoate, ethyl hexanoate, and isoamyl acetate are able to exert a strong influence on wine aroma: they are responsible for a major part of the aroma characteristics of young wines, but it does not mean they are the most significant aroma compounds in terms of sensory evaluation, and sensory studies are necessary to further confirm the impact of the odor-active compounds already identified. In contrast, the contributions of phenylethyl acetate and 2-phenylethanol were minimal. Based on their ROC, phenylethyl acetate and 2-phenylethanol each accounted for less than 1% of the global aroma perceptions of the three wines. According to Escudero *et al*. [32], even if they were present at a concentration higher than their threshold values, compounds such as fusel alcohols, acids, esters and volatile phenols are not able to affect individually the flavor of the wines. This is because these compounds are common in any kind of fermented alcoholic beverages that share similar aromatic properties. Moreover, the aromatic buffer would be caused by the presence in wine of relatively high concentrations of ethanol, ethyl esters, fusel alcohols, volatile phenols, *β*-damascenone, and fatty acids and can be broken only by the presence of an aroma with very different aroma properties, such as 4-methyl-4-mercaptopentan-2-one.

## 3. Experimental

### 3.1. Vineyard conditions and vinification

All vineyards are located in the Rongzi Chateau of Xiangning County in the Loess Plateau region, which is situated approximately between 35°59′–36°02′ N, 110°47′–110°50′ E with an altitude of 1,200–1,300 m, and where the loess depth exceeds 200 m. In the collection all the vineyards have similar characteristics (age and cultivation management). All the vines were cultivated in Spring 2007, in a seedling root system with multiple main vine fan-training, and 2.5 × 1.0 m (row × vine) spacing. 

All grape berries were harvested manually at optimum technological maturity, as judged by indices of sugar and acid content in 2009. Pre-fermentation treatments and winemaking were performed as described by Li [33]. Briefly, grapes were crushed on an experimental destemmer-crusher and then transferred to stainless-steel containers. Thirty L of each treatment wine were produced in three replicates. Fifty mg/L of SO_2_ and 30 mg/L of pectinase (Lallzyme Ex) were added to the musts and the contents were mixed by hand. After maceration of the musts for 24 h, 200 mg/L of dried active yeast (*Saccharomyces cerevisiae* strain, Lallemand, Danstar Ferment AG, Switzerland) was added to the musts, according to commercial specifications. Alcoholic fermentation was carried out at 20 to 25 °C to dryness (reducing sugar < 4 g/L) which took place over a 6–8 days period and density controls were maintained during this period. At the end of alcoholic fermentation the wines were separated from pomace, and then added 50 mg/L of SO_2_. After fermentation, the wine samples were bottled and stored at 5 °C prior to analysis. All the samples were five months old at the time of analysis. Total sugar, total acidity, pH, total phenolics, total tannins, reducing sugar and ethanol were analyzed [34] (Table 3).

**Table 3:** General composition of the three musts and wines.

	Cabernet Sauvignon		Cabernet Gernischet		Chardonnay	
	Must	Wine	Must	Wine	Must	Wine
**Total sugar (g/L)**	194.0	—	196.5	—	185.3	—
**Total acidity ^a ^(g/L)**	10.4	8.9	10.4	8.9	8.7	6.0
**PH**	3.1	3.0	3.1	3.1	3.7	3.4
**Total phenolics ^b^ (mg/L)**	3430	1152	2790	1193	1760	192
**Total tannins ^c ^(mg/L)**	4060	933	3490	1038	1850	191
**Reducing sugar (g/L)**	—	1.7	—	2.0	—	1.3
**Ethanol (%,v/v)**	—	11.8	—	12.2	—	12.7

The data were mean values of triplicate samples (maximum SD: ±10%); ^a^ Acidity expressed as grams of tartaric acid equivalents per liter; ^b^ Total phenolics expressed as milligrams of gallic acid equivalents per liter; ^c^ Tannins expressed as milligrams of tannin acid equivalents per liter.

### 3.2. Reagents

All standards were purchased from Aldrich (Milwaukee, WI, USA) and Fluka (Buchs, Switzerland). Purity of all standards was above 99%. Model solutions were prepared using the methods reported by Howard *et al*. [35]. 4-Methyl-2-pentanol was used as the internal standard. For quantification, 8-point calibration curves for each compound were prepared using the method described by Ferreira *et al*. [7], which was also used as a reference to determine the concentration range of standard solutions. The regression coefficients of calibration curves were above 98%.

### 3.3. HS-SPME procedure

Aroma compounds of the wine samples were extracted by HS-SPME and analyzed using gas chromatography/mass spectrometry as described by Zhang *et al*. [8]. Five milliliters of wine sample and 1 g NaCl were placed in a 15-mL sample vial. The vial was tightly capped with a PTFE-silicon septum and heated at 40 °C for 30 min on a heating platform agitation at 400 rpm. The SPME (50/30-*μ*m DVB/Carboxen/PDMS, Supelco, Bellefonte, PA, USA), preconditioned according to manufacturer’s instruction, was then inserted into the headspace, where extraction was allowed to occur for 30 min with continued heating and agitation by a magnetic stirrer. The fiber was subsequently desorbed in the GC injector for 25 min.

### 3.4. GC–MS analysis

The GC-MS system used was an Agilent 6890 GC equipped with an Agilent 5975 mass spectrometer. The column used was a 60 m × 0.25 mm HP-INNOWAX capillary with 0.25 μm film thickness (J & W Scientific, Folsom, CA, USA). The carrier gas was helium at a flow rate of 1 mL/min. Samples were injected by placing the SPME fiber at the GC inlet for 25 min with the splitless mode. The oven’s starting temperature was 50 °C, which was held for 1 min, then raised to 220 °C at a rate of 3 °C /min and held at 220 °C for 5 min. The mass spectrometry in the electron impact mode (MS/EI) at 70 eV was recorded in the range *m*/*z* 20 to 450 U. The mass spectrophotometer was operated in the selective ion mode under autotune conditions and the area of each peak was determined by ChemStation software (Agilent Technologies). Analyses were carried out in triplicate.

### 3.5. Statistical analysis

Statistical analysis was conducted by SPSS 16.0 for Windows with three replications of the same sample.

## 4. Conclusion

This work shows the first study on volatile compounds of young wines from Cabernet Sauvignon, Cabernet Gernischet and Chardonnay varieties grown in the Loess Plateau region of China. In this study, a total of 45, 44 and 42 volatile compounds were identified and quantified in Cabernet Sauvignon, Cabernet Gernischet and Chardonnay wines, respectively. The three young wines analyzed were characterized by the presence of higher levels of higher alcohols, esters and fatty acids. According to the OAVs and ROC values, 18 volatile compounds were always present in the three wines at concentrations higher than their threshold values, but ethyl octanoate, ethyl hexanoate and isoamyl acetate were the most characteristic aroma-active compounds of the three young wines. 
